# Interactions with successional stage and nutrient status determines the life-form-specific effects of increased soil temperature on boreal forest floor vegetation

**DOI:** 10.1002/ece3.1412

**Published:** 2015-01-30

**Authors:** Per-Ola Hedwall, Jerry Skoglund, Sune Linder

**Affiliations:** 1 Swedish University of Agricultural Sciences, SLU, Southern Swedish Forest Research CentrePO Box 49, SE-230 53, Alnarp, Sweden; 2 Department of Ecology, SLUSE-750 07, Uppsala, Sweden

**Keywords:** Eutrophication, global change, light restriction, *Picea abies*, soil temperature, understorey vegetation

## Abstract

The boreal forest is one of the largest terrestrial biomes and plays a key role for the global carbon balance and climate. The forest floor vegetation has a strong influence on the carbon and nitrogen cycles of the forests and is sensitive to changes in temperature conditions and nutrient availability. Additionally, the effects of climate warming on forest floor vegetation have been suggested to be moderated by the tree layer. Data on the effects of soil warming on forest floor vegetation from the boreal forest are, however, very scarce. We studied the effects on the forest floor vegetation in a long-term (18 years) soil warming and fertilization experiment in a Norway spruce stand in northern Sweden. During the first 9 years, warming favored early successional species such as grasses and forbs at the expense of dwarf shrubs and bryophytes in unfertilized stands, while the effects were smaller after fertilization. Hence, warming led to significant changes in species composition and an increase in species richness in the open canopy nutrient limited forest. After another 9 years of warming and increasing tree canopy closure, most of the initial effects had ceased, indicating an interaction between forest succession and warming. The only remaining effect of warming was on the abundance of bryophytes, which contrary to the initial phase was strongly favored by warming. We propose that the suggested moderating effects of the tree layer are specific to plant life-form and conclude that the successional phase of the forest may have a considerable impact on the effects of climate change on forest floor vegetation and its feedback effects on the carbon and nitrogen cycles, and thus on the climate.

## Introduction

The boreal area is one of the largest terrestrial biomes in the world (Melillo et al. 1993; Bartholomé and Belward 2005) and is characterized by low temperatures and high soil carbon storage because of low rates of turnover (Anderson 1991; Goulden et al. 1998). Forest is the most common boreal vegetation type and coniferous tree species commonly dominate. Low forest productivity results in open tree canopies and often abundant forest floor vegetation dominated by herbs and grasses on nutrient rich sites, but vast nutrient poor areas are covered by ericaceous dwarf shrubs, bryophytes, and lichens. Forest floor vegetation plays an important role in boreal forest ecosystems because of its influence on soil processes, nutrient cycling, litter decomposition, and forest succession (Nilsson and Wardle 2005; Sardans and Peñuelas 2012; Lindo et al. 2013). In the open northern boreal forests, the understorey also contributes a considerable part of the annual photosynthetic production (Kolari et al. 2006).

Boreal forests are in general nitrogen (N)-limited (Tamm 1991), and small changes in nutrient availability can cause changes in the relative abundance of the dominant species in the forest floor vegetation (Nordin et al. 2009). Increased soil temperature during summer is a likely effect of climate warming (Jungqvist et al. 2014), while a reduction in snow cover during winter could lead to lower soil temperature during winter (Kreyling et al. 2012). Several studies have reported increased N mineralization as a result of experimental soil warming (Rustad et al. 2001; Contosta et al. 2011; Sardans and Peñuelas 2012), which may cause subsequent changes in the forest floor vegetation.

Our understanding of the effects of increased soil temperature on composition and diversity of ground vegetation in northern biomes is mainly based on studies performed in tundra (Jónsdottir et al. 2005; Walker et al. 2006; Elmendorf et al. 2012), heath (Peñuelas et al. 2004; Michelsen et al. 2012), bog and fen (Weltzin et al. 2000), or grassland ecosystems (De Valpine and Harte 2001; Cantarel et al. 2012). A meta-analysis of warming effects on tundra vegetation has shown that deciduous shrubs and grasses increase in cover, while bryophytes decrease by increased temperatures (Walker et al. 2006). Elmendorf et al. (2012) suggested that the effects may be dependent on site characteristics such as ambient temperature and found little evidence for nonlinear trends in the warming effect over time in these open environments. The species composition in the subarctic tundra ecosystems may overlap considerably with that of northern boreal forest, but the composition and density of the tree layer has a strong influence on the forest floor vegetation (Hart and Chen 2006, 2008; Hedwall et al. 2013a), as well as the response to nutrient enrichment (Verheyen et al. 2012; Hedwall et al. 2013b), and climate change as the tree layer may moderate the effects of higher temperatures (De Frenne et al. 2013). Early successional stages of these forests are characterized by rapid changes in species composition and relative abundances of species, but as the forest ages, and in the absence of disturbance, plant communities become more stable (Widenfalk and Weslien 2009). Hence, the effects of soil warming may be dependent on successional stage of the forest, implying nonlinear changes in effect over time, which emphasize the need of long-term studies (De Frenne et al. 2013). There are reasons to believe that, despite the commonalities with tundra vegetation, the effects of increased soil temperature may be different in boreal forests with a dynamic tree layer than in the open tundra. Unfortunately, the available studies on forest floor vegetation are mainly of short duration, which makes it unlikely to catch the long-term effects of changes in tree canopy cover, or from other biomes than the boreal (Farnsworth et al. 1995; Dawes et al. 2011).

This study was performed in a long-term soil-warming experiment (Bergh and Linder 1999) established in the Flakaliden nutrient optimization experiment in northern Sweden (Linder 1995). The warming experiment, one of the longest running in a forest ecosystem, consisted of both fertilized and unfertilized replicated 100 m^2^ plots. The objective of this study was to follow the long-term effects of increased soil temperatures on the composition and diversity of the forest floor vegetation, and to improve our comprehension of boreal forest ecosystems under climate change. We analyzed data annually on the composition of the forest floor vegetation for the first 9 years (1994–2002), and a final inventory was made 9 years later (2011).

Fertilization has been shown to have strong effects on the forest floor vegetation in this type of forest, decreasing the abundance of many species while increasing only a few nitrophilic species, which can persist in low light environments (Hedwall et al. 2010, 2011). We hypothesized that early successional species would be favored by soil warming at the expense of N-conservative species such as ericaceous dwarf shrubs. Furthermore, we expected the dominant bryophytes to be disfavored by soil warming because of the positive effects on more competitive vascular plants, but only small additional effects in fertilized stands with already high nutrient availability.

## Material and Methods

### Site description

The study was performed in a long-term nutrient optimization experiment at Flakaliden (64°07′N; 19°27′E; alt. 310 m a.s.l.) in northern Sweden. The experiment was established in 1986 in a young stand of Norway spruce (*Picea abies*), planted in 1963, after prescribed burning and soil scarification, with 4-year-old seedlings of a local provenance. At the time of establishment, stand density was ∽2400 trees ha^−1^ with no subsequent thinnings. The forest floor vegetation is dominated by dwarf shrubs from the *Vaccinium* genus and the bryophytes *Hylocomium splendens* and *Pleurozium schreberi*.

The site belongs to the middle boreal subzone (Sjörs 1999) and has a harsh boreal climate with long cool days in the summer and short cold days in the winter. The mean annual air temperature is 2.4 °C, and the mean monthly temperature varies from −7.5 °C in February to 14.6 °C in July (mean for 1990–2009). Mean annual rainfall is ∽600 mm with approximately one-third falling as snow, which usually covers the frozen ground from mid-October to early May. The length of the growing season (the period with a daily mean air temperature ≥ +5°C) averages ∽150 days, but with large between-year variations (Sigurdsson et al. 2013).

The soil at the site is a thin podzolic, sandy, postglacial till with mean depth of about 120 cm, with a 2–6 cm thick humus layer, and with soil water content normally not limiting for tree growth (Bergh et al. 1999). The site fertility is relatively low (the dominant height at age 100 years, *H*_100_ = 17–19 m; Hägglund and Lundmark 1977).

### Treatments

The nutrient optimization treatments, which began in 1987, included untreated control plots, irrigation, and two nutrient optimization treatments (Linder and Flower-Ellis 1992). In this study, only irrigated (I) and irrigated-fertilized (IL) plots were included. The reason for using treatments including irrigation was to reduce the risk of drying the soil as an effect of warming. In the IL treatment, all essential macro- and micronutrients were supplied every second day during the growing season (mid-June to mid-August), and water was supplied to the plots to maintain a soil water potential above −100 kPa. The amount of nutrient elements supplied to the IL plots, before (1987–1994) and during (1995–2011) this study is given in Table1. Nitrogen deposition in the region averages 3 kg N·ha^−1^·a^−1^ (Berggren et al. 2004) and net nitrogen mineralization is 4 and 18 kg N·ha^−1^·a^−1^ in control and fertilized stands, respectively (Andersson et al. 2002). For further details on experimental design and treatments, see Linder (1995) and Bergh et al. (1999).

**Table 1:** Amounts of macro- and micronutrient elements (kg·ha^−1^) supplied to irrigated-fertilized (IL) stands at Flakaliden, during the period 1987–1994, and 1995–2011, respectively. For further details regarding treatments, see Linder (1995)

Period	N	P	K	Ca	Mg	S	Mn	Fe	Zn	B	Cu
1987–1994	675	115	327	68	58	28	2.8	4.8	0.2	3.4	0.2
1995–2011	925	156	420	65	83	43	1.8	2.8	0.2	1.6	0.1
Total	1600	271	747	133	141	71	4.6	7.6	0.4	5.0	0.3

During the summer of 1994, a soil-warming treatment was installed in the buffer zone of one irrigated (I) and one irrigated-fertilized (IL) stand, with two 10 × 10 m subplots per treatment. This implies that there is no true replication of the fertilization treatment, and this factor is included in the analyses only to test the effects of warming in different environmental settings. Each heated subplot (h) had an unheated control plot (c), hereafter referred to as Ic, Ih, ILc, and ILh, respectively. The number of trees per subplot varied between 21 and 28, but the basal area per plot was initially similar within each treatment. At the time when the soil-warming experiment was initiated, the unfertilized stands were still successionally young with low stem volumes (∽33 m^3^·ha^−1^) and open canopies, but the fertilized stands had responded strongly to the optimized fertilization and had a volume of 85 m^3^·ha^−1^ and canopy cover of ∽80% (Bergh and Linder 1999; Bergh et al. 2005). The volume increased linearly in both the control and the fertilized treatments during the study period, maintaining the relative relation between the treatments (Hedwall et al. 2013b). All plots were fenced to exclude animals, and boardwalks were installed to prevent trampling of the ground vegetation.

The design of the soil-warming treatment followed in principle the system described by Peterjohn et al. (1993). Six, 85-m long, heating cables (DEVI Elektrovärme AB, Vällingby, Sweden) per subplot were buried under the humus layer at a spacing of *ca*. 20 cm. After cutting through the moss- and humus layer with a knife, the cables were placed on the mineral soil, after which the furrows were closed. The control plots were disturbed in the same way as the warmed plots, but heating cables were not installed. The heating system was controlled and monitored by temperature sensors connected to a data logger (Campbell CR10; Campbell Scientific Inc., Logan, UT). On each plot (heated and control plots), six thermocouples were installed into the first centimeter of the mineral soil. These sensors were monitored and data stored every 15 min.

From 1995 to 2011, soil warming started in early April each year, about 5 weeks before the soil thawed in the unheated plots. The soil temperature was increased 1 °C per week, until a 5 °C difference between heated and control plots was reached. In late autumn, when the soil temperature in the control plots approached 0 °C, the soil temperature of the heated plots was decreased by 1 °C per week. If the control plots did not freeze before 1 November, the temperature reduction was still initiated. For further information on the construction and long-term performance of the soil-warming system, see Bergh and Linder (1999) and Strömgren and Linder (2002).

### Vegetation inventory

The forest floor vegetation was surveyed annually from before the start of the warming experiment in 1994 until 2002; a final inventory was made in 2011. The cover of plant species was estimated individually in one percent classes in nine permanent 0.5 m × 0.5 m squares per plot. The total cover of bryophytes was also recorded. The average cover of individual species and groups of species within plots was then calculated and used in the following analyses. The plants were, if possible, determined to species level. Nomenclature used in this paper is according to the Swedish Taxonomic Database – Dyntaxa (Anonymous 2012).

### Data analyses

All statistical analyses were performed in R 3.0.1 (R Development Core Team 2013). Data from 1994 to 2002 concerning the abundance of *Avenella flexuosa*, *Vaccinium myrtillus,* and *V. vitis-idaea,* which were dominating nonwarmed and unfertilized plots, were analyzed for differences as a result of warming and time since warming started, and their interaction using generalized linear mixed models (GLMM). The total cover of bryophytes and forbs, as well as species richness and Shannon's diversity, was also analyzed by GLMMs. Warming was treated as factor variable and time since warming started as a continuous variable. The time variable started from zero which implies that a significant effect of warming in the full model, including the two main terms and their interaction, indicates initial differences between the warmed and nonwarmed plots. Data constituted repeated measurements on the same object, and hence plot was introduced as a random variable in the GLMMs. Generalized additive mixed models (GAMM) with plot as random variable were applied to test whether there were nonlinear patterns in the trends in the response variables along the time axes. A GAMM is a generalization of a GLMM in which the assumption of a linear relationship between predictors and the (transformed) response variable is relaxed (Wood 2006). The GAMMs were built without prior assumptions about the shape of the species–environment relationship between the variables. GAMMs add smooth components to a linear model and therefore, in principle, no restriction exists in the shapes that they can take. To avoid overfitting, model complexity was restricted by applying generalized cross-validation, to optimize the smoothing parameter. All species and groups of species were modeled with a gamma distribution and log-link. The GLMMs were made with the *glmmpql* function in the MASS package (Venables and Ripley 2002) and the GAMMs in the *mgcv* package (Wood 2006). In addition to the GLMM data, all response variables from 2011 were modeled as effects of fertilization and warming together with the interaction to check for remaining effects at the end of the observation period. This was performed by applying generalized linear models (GLM) as implemented in the *glm* function in the standard version of R. The same distributions and links as in the GLMMs were used for the GLMs. For each model, the residuals were plotted against the fitted values to check for heteroscedasticity and remaining patterns. Final models were determined by a partial backward selection procedure with *P* = 0.05 as threshold for inclusion of variables, removing nonsignificant interactions. Tests of partial correlation, controlling for the effects of the treatments, between the response variables used in the GLMMs, and basal area were performed to check for effects of canopy closure over the period 1994–2011.

To test for plant community response to warming and time since warming started, a permutational multivariate analysis of variances (PERMANOVA) with Bray–Curtis distance, as described by Anderson (2001) and implemented in the *adonis* function in the Vegan package in R (Oksanen et al. 2011), was performed on the 1994–2002 data and 2011 data separately. The PERMANOVA was performed with 999 permutations, which were constrained within plot for the 1994–2002 data. Additionally, vascular species richness and Shannon's diversity were calculated by the *specnumber* and *diversity* functions in the Vegan package.

To identify species that characterized the different treatments, indicator species analyses (Dufréne and Legendre 1997) were performed with warming as grouping variable within the fertilization treatment by the *indval* function in the R package *labdsv* (Roberts 2013).

## Results

**Figure 1:**
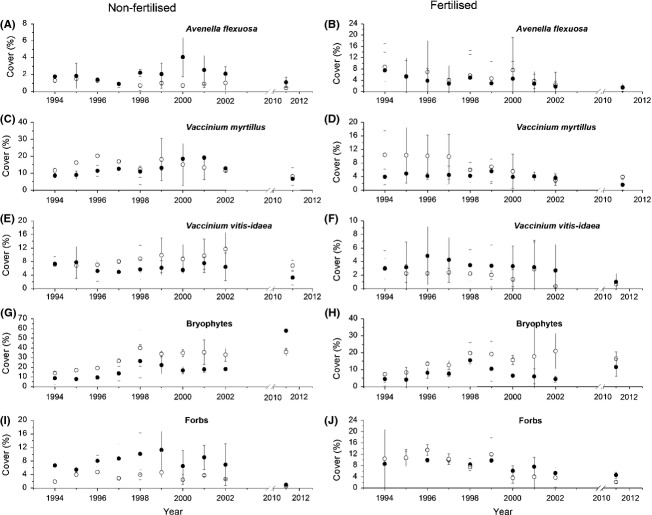
Cover (%) of *Avenella flexuosa* (A, B), *Vaccinium myrtillus* (C, D), *V. vitis-idaea* (E, F), bryophytes (G, H), and forbs (I, J) in 1994–2002 and in 2011. Left column is unfertilized plots (Ic & Ih) and the right column is fertilized plots (ILc & ILh). Symbols: Nonheated plots (open circles); heated plots (filled circles). Error bars indicate ± 2SE.

As indicated by the lack of significant effects of warming when the interaction between warming and time was included in the GLMMs, there were no indications of initial differences between warmed and nonwarmed plots within fertilization treatment in any of the response variables except *V. myrtillus* in fertilized plots (Table2; Fig.1). Between 1993 and 2011, the basal area of the forest increased linearly from 7 to 24 m^2^·ha^−1^ in nonfertilized plots and from 13 to 46 m^2^·ha^−1^ in fertilized plots. There were significant negative correlations between the basal area and *A. flexuosa* (corr = −0.455; *P* < 0.001), *V. myrtillus* (corr = −0.338; *P* = 0.003), forbs (corr = −0.638; *P* < 0.001), species richness (corr = −0.369; *P* = 0.001), Shannon diversity (corr = −0.415; *P* < 0.001), and *V. vitis-idaea* (corr = −0.227; *P* = 0.052), although the last-mentioned was only borderline significant. The total cover of bryophytes, on the other hand, increased with basal area (corr = 0.412; *P* < 0.001).

**Table 2:** Generalized linear mixed models (GLMM) for cover of individual species and groups of species, as well as vascular species richness and Shannon's diversity from the start of the warming experiment in 1994 until 2002. The GLMMs were performed separately for the fertilized and non-fertilized plots with plot as a random variable. A partial model selection was done, removing nonsignificant interaction terms (*P *>* *0.05)

			Warming		Time		*W* × *T*	
	Species	Model	*t*	*P*	*t*	*P*	*t*	*P*
Non-fertilized	*Avenella flexuosa*	Full	1.1	0.390	−0.7	0.520	1.9	0.074
		Reduced	3.2	0.085	1.2	0.236		
	*Vaccinium myrtillus*	Full	−1.4	0.301	0.3	0.752	2.4	0.024
	*Vaccinium vitis-idaea*	Full	−1.0	0.408	1.3	0.209	−1.2	0.233
		Reduced	−1.7	0.226	0.481	0.635		
	Bryophytes	Full	−2.6	0.124	2.5	0.020	0.4	0.679
		Reduced	−4.1	0.054	4.5	<0.001		
	Forbs	Full	2.2	0.154	−0.7	0.461	1.0	0.306
		Reduced	4.4	0.047	0.1	0.908		
	Species richness	Full	−1.6	0.256	−3.8	<0.001	3.6	0.001
	Shannon's diversity	Full	2.9	0.101	−1.1	0.276	0.2	0.809
		Reduced	6.8	0.021	−1.5	0.150		
Fertilized	*Avenella flexuosa*	Full	−0.4	0.722	−3.0	0.006	0.0	0.968
		Reduced	−0.4	0.702	−4.3	<0.001		
	*Vaccinium myrtillus*	Full	−5.0	0.037	−5.1	<0.001	3.3	0.002
	*Vaccinium vitis-idaea*	Full	0.2	0.863	−2.7	0.011	1.3	0.199
		Reduced	1.0	0.409	−2.6	0.013		
	Bryophytes	Full	−0.7	0.534	2.3	0.027	−1.4	0.174
		Reduced	−3.5	0.072	2.1	0.047		
	Forbs	Full	1.4	0.304	−2.0	0.054	−2.0	0.057
		Reduced	−0.5	0.652	−4.7	<0.001		
	Species richness	Full	1.3	0.326	−1.6	0.115	1.4	0.178
		Reduced	4.5	0.047	−0.9	0.380		
	Shannon's diversity		2.7	0.116	0.1	0.947	−2.3	0.030

**Table 3:** A Generalized linear model (GLM) for cover of individual species and groups of species, as well as vascular species richness and Shannon's diversity in 2011. A partial model selection was done, removing nonsignificant interaction terms (*P *>* *0.05)

Species	Model	Fertilization		Warming		*F* × *W*	
		*t*	*P*	*t*	*P*	*t*	*P*
*Avenella flexuosa*	Full	2.4	0.074	1.9	0.126	−1.3	0.278
	Reduced	2.0	0.108	1.3	0.237		
*Vaccinium myrtillus*	Full	−1.8	0.150	−0.5	0.673	−1.2	0.297
	Reduced	−4.1	0.010	−2.0	0.100		
*Vaccinium vitis-idaea*	Full	−3.8	0.020	−1.2	0.310	1.3	0.265
	Reduced	−3.6	0.015	−0.3	0.767		
Bryophytes	Full	−7.1	0.002	8.0	0.001	−6.9	0.002
Forbs	Full	9.4	0.001	1.0	0.376	−4.8	0.009
Species richness	Full	0.3	0.749	1.4	0.242	−0.7	0.507
	Reduced	−0.3	0.809	1.3	0.259		
Shannon's diversity	Full	3.2	0.034	2.4	0.074	−1.5	0.217
	Reduced	2.7	0.042	1.7	0.142		

**Figure 2:**
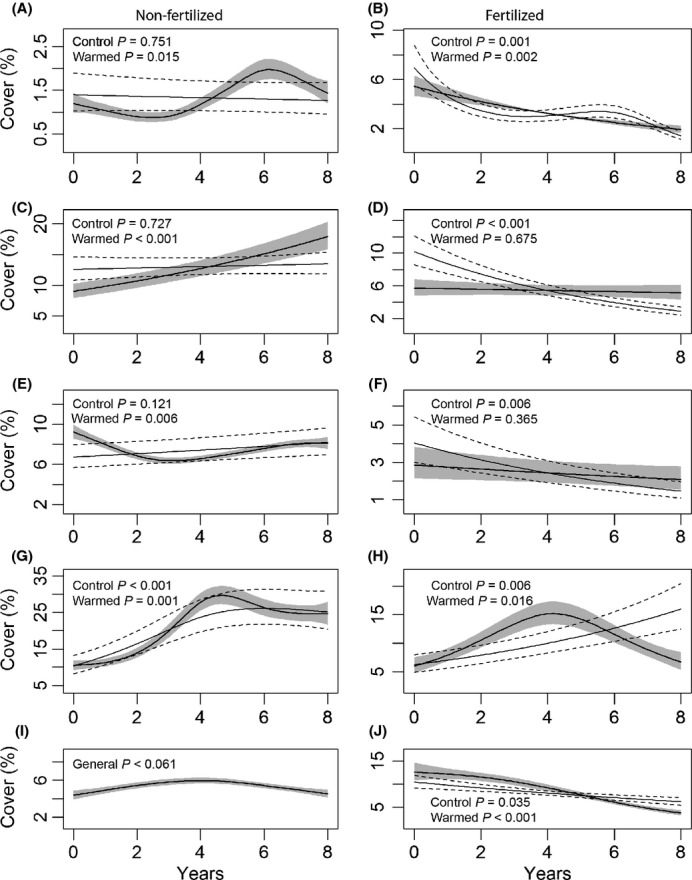
Generalized additive mixed models (GAMM) of the development of the cover (%) of *Avenella flexuosa* (A, B), *Vaccinium myrtillus* (C, D), *V. vitis-idaea* (E, F), bryophytes (G, H), and forbs (I, J) from 1994 to 2002. Left column is unfertilized plots (Ic & Ih) and the right column is fertilized plots (ILc & ILh). Error bars indicate ± 1SE. The GAMMs should only be internally compared concerning their shape and slope, and not concerning their relative location along the *y*-axis as the warming factor was not included in the model.

The GLMMs for *A. flexuosa* showed no significant response to warming in either fertilized or nonfertilized plots. This species decreased in cover by time in fertilized plots, while the GLMM revealed no linear patterns along the time axis in nonfertilized plots (Table2; Fig.1A and B). However, the GAMM for warmed nonfertilized plots showed a significant trend with an increase in cover after 3–4 years, a trend that was absent in nonwarmed plots (Fig.2A and B).

There was a significant interaction between warming and time in nonfertilized plots in the GLMM of the cover of *V. myrtillus*, indicating a difference in development as a result of warming (Table2; Fig.1C and D). This was confirmed by the GAMM in warmed plots which, in contrast to nonwarmed, showed an increase in cover by time (Fig.2C and D). There were no significant effects of warming on *V. vitis-idaea* independently of fertilization. The cover decreased with time in fertilized plots according to the GLMM (Table2; Fig.1E and F), which was also seen in the GAMMs. The GAMM for warmed nonfertilized plots revealed a significant decrease in cover during the first 2 to 3 years, an effect that later levelled off (Fig.2E and F).

Warming resulted in near-significant negative effects on the bryophytes in nonfertilized plots (Table2). The bryophytes increased in cover during the study period independent of treatment (Table2; Fig.1G and H). In warmed fertilized plots, the GAMM indicated a mid-term peak in cover with a following decrease (Fig.2G and H). In nonfertilized plots, the forbs increased in cover as an effect of warming. While there was no significant trend on the cover of forbs in nonfertilized plots, the cover decreased with time in fertilized plots independent of warming (Table2; Figs.1I,J and 2I,J).

**Figure 3:**
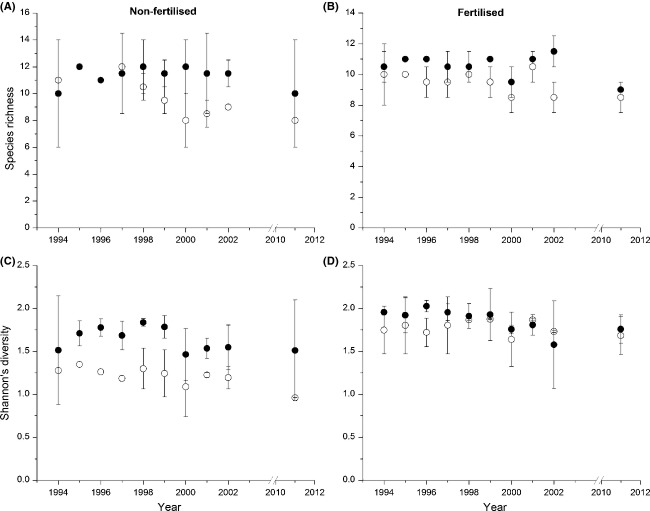
Vascular plant species richness and Shannon's diversity in 1994–2002 and in 2011. Left column is unfertilized plots and the right column is fertilized plots. Symbols: Nonheated plots (open circles); heated plots (filled circles). Error bars indicate ± 2SE.

There was a significant interaction between warming and time, and a decreasing trend over time in nonfertilized plots, on species richness according to the GLMM (Table2; Fig.3A and B). This indicates a decrease in number of species in nonwarmed plots and a difference in development because of warming. Likewise, there was a positive effect of warming on the number of species in fertilized plots. Warming increased Shannon's diversity in nonfertilized plots, while there were differences in trends over time between warmed and nonwarmed plots in fertilized plots. Here, the Shannon's diversity decreased in warmed plots, while it was stable in nonwarmed (Table2; Fig.3C and D).

In 2011, according to the GLMs, the only remaining effect of warming was a significant increase in the cover of bryophytes in unfertilized stands (Fig.1G), while there were significant effects of fertilization on all response variables except *A. flexuosa* and species richness (Table3). The most important factor explaining the composition of the plant community during 1994–2002 was warming, independently of fertilization, with *r*^2^ = 0.27 and *r*^2^ = 0.14 for the nonfertilized and fertilized plots, respectively in the PERMANOVA (Table4). The composition changed significantly over time and the significant interactions between warming and time indicates that this change differed depending on if warming was applied. In 2011, the only significant factor for the plant community was fertilization, with *r*^2^ = 0.53. The indicator species analyses of the 1994–2002 data showed significant species-specific differences in the results of warming depending on fertilization. In nonfertilized plots, the bryophyte *P. schreberi* (−62%) and the dwarf shrubs *V. vitis-idaea* and *Linnea borealis* indicated nonwarmed conditions, while all indicators of warmed plots were grasses, forbs, and ferns. The forbs with the highest indicator values were *Epilobium angustifolium*, *Melampyrum sylvaticum,* and *Maianthemum bifolium* (Table5). Also in fertilized plots, most indicators of nonwarmed conditions were bryophytes and dwarf shrubs, while forbs and grass increased by warming. There were two exceptions to this pattern. First, the fern *Gymnocarpium dryopteris* had a much lower abundance in warmed plots than in nonwarmed; second, the cover of *V. vitis-idaea* was higher under warmed conditions (Table5). While the specific species indicating nonwarmed conditions were different depending on fertilization, there was a considerable overlap in the indicators for warmed plots. Of the nine species that were significant indicators of warming *vs*. nonwarming in nonfertilized plots in Table5, three and six were indicators of nonwarmed and warmed plots, respectively, indicating an increase in number of species. In contrast, the indicators were evenly distributed between winners and losers in fertilized plots, suggesting increased species turnover.

**Table 4:** Permutational MANOVA (PM) with 999 permutations of the plant species community and warming, time since the start of warming and their interaction as covariates for the period 1994–2002. The permutations were constrained within plot. The lower part shows a PM of the plant species community in 2011 with fertilization, warming and their interaction as covariates

	*F*	*r* ^2^	*P*
1994–2002			
Nonfertilized			
Warming	14.6	0.27	0.001
Time	8.3	0.16	0.001
*W* × *T*	4.5	0.08	0.001
Fertilized			
Warming	6.4	0.14	0.001
Time	5.5	0.12	0.001
*W* × *T*	1.3	0.03	0.021
2011			
Fertilization	7.0	0.53	0.016
Warming	0.8	0.06	0.530
*F* × *W*	1.4	0.11	0.277

**Table 5:** Indicator species analyses within fertilization treatment and average cover by combination of treatment between 1994 and 2002. Only significant (*P *≤* *0.05) species is shown

						Cover (%)	
	Species	Growth form	Treatment	Indicator value	*P*	Control	Warmed
Nonfertilized	*Pleurozium schreberi*	Bryophyte	Ic	0.7219	0.002	19.0	7.3
	*Vaccinium vitis-idaea*	Dwarf shrub	Ic	0.5956	0.002	9.0	6.1
	*Linnea borealis*	Dwarf shrub	Ic	0.4683	0.045	0.1	0.0
	*Epilobium angustifolium*	Forb	Ih	0.9265	0.001	0.1	1.5
	*Melampyrum sylvaticum*	Forb	Ih	0.8072	0.001	0.0	0.5
	*Maianthemum bifolium*	Forb	Ih	0.7573	0.001	0.9	2.9
	*Luzula pilosa*	Grass	Ih	0.6898	0.001	0.4	0.8
	*Avenella flexuosa*	Grass	Ih	0.6869	0.001	1.0	2.1
	*Hieracium* sp	Forb	Ih	0.5192	0.005	0.0	0.1
	*Gymnocarpium dryopteris*	Fern	Ih	0.5000	0.004	0.0	0.2
Fertilized	*Gymnocarpium dryopteris*	Fern	ILc	0.9444	0.001	2.6	0.0
	*Dicranum* sp	Bryophyte	ILc	0.6984	0.011	1.9	0.7
	*Vaccinium myrtillus*	Dwarf shrub	ILc	0.6303	0.001	7.4	4.3
	*Hylocomium splendens*	Bryophyte	ILc	0.5556	0.001	0.2	0.0
	*Rhodobryum roseum*	Bryophyte	ILc	0.3889	0.005	0.2	0.0
	*Epilobium angustifolium*	Forb	ILh	0.8064	0.001	0.5	2.0
	*Melampyrum sylvaticum*	Forb	ILh	0.6844	0.007	0.2	0.5
	*Vaccinium vitis-idaea*	Dwarf shrub	ILh	0.6267	0.008	2.1	3.5
	*Hieracium* sp	Forb	ILh	0.5963	0.001	0.0	0.2
	*Calamagrostis* sp	Grass	ILh	0.4485	0.009	0.0	0.1

## Discussion

A significant change in species composition and individual species abundances as an effect of soil warming was found during the first observation period of 9 years. The positive effect on grasses and forbs and reduction of the abundance of bryophytes and the dwarf shrub *V. vitis-idaea* were in accordance with our expectations. However, when the experiment was revisited after another 9 years of warming, the only remaining effect was on the total cover of bryophytes, which now instead had increased, indicating that the effect of warming may change over time. The effects of warming were generally smaller where nutrient availability had been increased by fertilization. The negative effect on bryophytes is in line with the results of Van Wijk et al. (2003) who related this to increased shading by taller plants and increased litterfall and suggested that the effects of soil warming may change over time. Accordingly, after another 9 years, this negative effect had turned positive in unfertilized plots (Ih). In 1994, when the warming experiment was initiated, the unfertilized stands at Flakaliden were still relatively open, with limited light restrictions for the forest floor vegetation. Initially, forbs, *V. myrtillus* and the grass *A. flexuosa* increased in abundance as a result of warming, implying increased competition for the low-growing bryophytes and suppressing a potential positive response to warming in this group of plants. Along with the succeeding crown expansion of the trees, vascular plants became more restricted by low light and the bryophytes, with lower light demand, could increase in cover independently of treatment which is in accordance with studies of general dynamics in boreal forest (Hart and Chen 2006, 2008; Hedwall et al. 2013a), and also increase as a response to warming. The tree layer has recently been shown to moderate the effects of both climate warming and N deposition on the forest floor vegetation (Verheyen et al. 2012; De Frenne et al. 2013). Our results support this and indicate, additionally, that this effect may be specific to life-form and that effects of warming on certain groups of plants may increase with increased cover of the trees.

Plant diversity has previously been shown to decrease or be unaffected by warming (Walker et al. 2006). In our study, surprisingly, soil warming led to higher species richness and Shannon's diversity. In plant communities dominated by dwarf shrubs, early successional stages are commonly, as an effect of light and nutrient availability, the most species rich (Hart and Chen 2006). Disturbance disfavors N-conservative ericoid species (in our case *Vaccinium* species) and bryophytes such as *P. schreberi* (Bergstedt et al. 2008) and may thus lead to more equal abundances (higher evenness) and, as in our case when associated with an increase in species richness, increased Shannon's diversity. When these young forests age, dwarf shrubs recover in abundance (Hedwall et al. 2013a), and species richness and diversity decline (Hart and Chen 2006; Widenfalk and Weslien 2009). In our experiment, this decline was delayed in heated and unfertilized plots, resulting in the positive effect of warming. However, in 2011, no significant effects of warming remained and all treatments tended to have decreased in species richness.

There are at least three possible reasons for why the effects of warming in most cases were smaller in fertilized than in unfertilized stands: (1) the potential effect of increased nutrient mineralization may be smaller in fertilized stands; (2) the abundance of the forest floor vegetation was strongly reduced by fertilization, implying smaller possible effect sizes; and (3) the reduction in light on the forest floor as an effect of increased canopy cover strongly reduced the possibilities of understorey plants to utilize an increased nutrient supply for growth. None of these reasons exclude each other, but it has been suggested that the effects of increased nutrient availability on boreal forest floor vegetation are strongly mediated by both competition within strata (Strengbom et al. 2004) and amensalism from the tree layer (Thomas et al. 1999; Strengbom and Nordin 2012). Additionally, when the warming experiment was commenced, the optimized fertilization experiment had been ongoing for several years, and the trees and understorey were already significantly affected (Bergh et al. 1999), which most likely contributed to this difference in effect of warming.

The strong effect of the tree layer on the forest floor vegetation is consistent over large amplitude of forests, including both natural and managed ecosystems (Hart and Chen 2006, 2008). However, although the site in this study represents common vegetation and soil types, our comprehension of soil-warming effects on forest floor vegetation would benefit considerably from knowledge acquired in other environmental settings. Jungqvist et al. (2014) projected that summer soil warming may be considerable without an increase in soil temperature during winter if the ground is covered by snow. A lack of snow cover may, however, induce increased soil frost and significantly affect the boreal forest floor vegetation (Kreyling et al. 2012). Likewise, may extreme warm periods damage the vegetation (Bokhorst et al. 2009) and thus changes in winter climate can mediate the effects of summer warming, which needs to be taken into account when estimating the large-scale effects of higher temperatures.

Even though the bryophytes were favored by increased canopy cover independently of treatment, the cover was lower on fertilized plots than on the control plots. This is probably a direct effect of the fertilization that has been observed in previous experiments (e.g., Hedwall et al. 2010). Bryophytes in general have a strong influence on C- and N-cycles of boreal forest because of their often large abundance, considerable share of the net primary production, recalcitrant litter, and associations with N-fixing bacteria. The boreal forest generally has low N input through deposition, and fixation by these associations may be the main pathway for N to enter the system (DeLuca et al. 2002). For example, have fixation rates of up to 2 kg·N·ha^−1^ year^−1^ been observed for the most abundant bryophyte here, *P. schreberi* (Lindo et al. 2013), a species that responded strongly to warming. Several factors such as temperature and moisture have been shown to control this process (Gundale et al. 2012a,b), but the relation between N-fixation rates and bryophyte abundance is clearly positive (Sorensen et al. 2012). The positive long-term effects of warming on bryophytes may thus have important implications for our understanding of both impacts of increased temperatures on the N-cycle of boreal forests and the feedbacks of global warming on the C-cycle.

As the effects of warming differed between stages of forest succession, changes in distribution of successional stages may affect the total effects of global warming on the forest floor vegetation as well as the associated feedback effects. Climate change has been predicted to, depending on region, increase frequencies of natural disturbances such as wildfires, gales, pests, and pathogens (Dale et al. 2001). Additionally, there may be interactions between disturbance legacies and climate change on the ecosystem response (Bond-Lamberty et al. 2014). It has, however, historically often been the interaction between changes in human land use and climatic changes that has elicited large-scale changes in boreal vegetation (Chapin et al. 2004). A large share of the boreal forest is managed for timber production with rotation periods decided by economical and operational constraints and an increase in growth may lead to decreasing rotation periods. If the forecasts of increased frequency of natural disturbances become true, along with decreasing rotation periods in managed forests, the share of forest in early successional stages will increase and influence the effects of increased temperatures on the vegetation and the associated feedbacks.

In conclusion, the results of this study stress the importance of long-term studies to reveal the effects of soil warming on plant communities. It shows that effects from studies in open environments, although with similar species assemblages, cannot be directly extrapolated to forests. In the beginning of the study period, the stands in our experiment were relatively open, and grasses and forbs were significantly favored by warming at the expense of bryophytes. After 18 years of warming, however, the only significant remaining effects of warming were on the cover of bryophytes, which now were clearly favored by increased temperatures. Our results suggest that the effects of warming may be dependent on site fertility and successional stage of the forest, and consequently both natural and anthropogenic disturbances are of importance for the effects of climate change on forest floor vegetation and its impact on N- and C-cycles. Additionally, we suggest that the moderating effect of the tree layer on forest floor vegetation may be specific to species life-form and that the tree layer may facilitate the effects of warming on bryophytes. Hence, changes in natural disturbance frequency and intensity will have implications for the direct effects of warming on the forest floor vegetation. Likewise, will management decisions concerning rotation length and density of the forest have considerable impacts on the outcome of warming.
